# Acute Kidney Injury Epidemiology in pediatrics

**DOI:** 10.1590/2175-8239-JBN-2018-0127

**Published:** 2018-11-14

**Authors:** Thais Lira Cleto-Yamane, Conrado Lysandro Rodrigues Gomes, Jose Hermogenes Rocco Suassuna, Paulo Koch Nogueira

**Affiliations:** 1 Universidade do Estado do Rio de Janeiro Rio de JaneiroRJ Brasil Universidade do Estado do Rio de Janeiro, Rio de Janeiro, RJ, Brasil.; 2 Universidade Federal de São Paulo São PauloSP Brasil Universidade Federal de São Paulo, São Paulo, SP, Brasil.

**Keywords:** Renal Failure, Acute Kidney Injury, Epidemiology

## Abstract

We performed a search in the MEDLINE database using the MeSH term: "Acute Kidney Injury", selecting the subtopic "Epidemiology", and applying age and year of publication filters. We also searched for the terms: "acute renal failure" and "epidemiology" "acute tubular necrosis" and "epidemiology" in the title and summary fields with the same filters. In a second search, we searched in the LILACS database, with the terms: "acute renal injury", or "acute renal failure" or "acute kidney injury" and the age filter. All abstracts were evaluated by the authors and the articles considered most relevant, were examined in their entirety. Acute Kidney Injury (AKI) -related mortality ranged from 3-63% in the studies included in this review. AKI etiology has marked regional differences, with sepsis being the main cause in developed countries. In developing countries, primary renal diseases and hypovolemia are still a common cause of AKI.

## Introduction

Acute kidney injury (AKI) is defined as the sudden reduction in renal function that can range from discrete changes in biochemical markers to kidney failure requiring artificial renal support (ARS). It is a severe complication, with high morbidity and mortality in critically-ill patients, and it is often of multifactorial etiology.^1^

Until the early 2000s, the absence of standardization for diagnosis, with the existence of more than 30 published definitions for AKI,^2^ made it impossible to determine its magnitude, as well as the comparison between the different studies on the subject. The most widely available studies deal primarily with AKI requiring ARS, reporting mortality rates between 11% and 63% in pediatric patients. Children who presented with AKI had longer periods of hospitalization and stayed in a pediatric intensive care unit (PICU), and had a greater need for mechanical ventilation.^5^^,^^6^ In addition, children surviving an episode of AKI may progress with chronic kidney disease (up to 60% of children remain with proteinuria, hypertension, and some degree of reduced glomerular filtration rate (GFR)).^7^^-^9

The need for uniformity in the definition of AKI resulted in the creation of the first standardized definition published in 2004, called RIFLE (Risk, Injury, Failure, Loss, End-stage). Three years afterwards, these criteria were adapted to the pediatric population, giving rise to the pediatric RIFLE (pRIFLE) acronym.^10^ Since then, this criterion has undergone two other modifications, the most recent one being the KDIGO (Kidney Disease: Improving Global Outcome) rating system published in 2012.^11^

ARS is the most effective treatment for severe AKI in critically-ill patients. The first reports of hemodialysis (HD) use in humans date back to the 1940s, when Kolff et al. described the use of the so-called "artificial kidney" in a 29-year-old woman with chronic glomerulonephritis and uremia.^12^ In 1957, the same author described the advances in the equipment developed until then and mentioned the indications for using the artificial kidney, including AKI secondary to shock. The use of HD in children required more time to be implemented due to persistent difficulties, such as the small vessel size in pediatric patients and the need for large volumes to fill the extracorporeal circuit.^14^ In 1957, the first report of HD cases in children was published, describing clinical improvement and greater ease in conservative management after its use in 5 five children from 2 to 14 years of age.^14^ However, this modality has not proved safe for infants and young children. In this context, Segar et al. described the importance of using peritoneal dialysis in patients under 1 year of age and/or under 15 kg.

From the earliest reports of AKI to the present day, there has been significant improvement in the techniques and availability of dialysis procedures, as well as dramatic changes in epidemiology, especially regarding the causes of AKI. Primary renal diseases predominated, such as acute glomerulonephritis and hemolytic-uremic syndrome. After the advent of intensive care, sepsis, large surgeries (such as cardiac surgeries) and oncological problems became the most frequent causes,^16^^,^^17^ although in developing countries, dehydration is still a relevant cause of AKI.^18^^-^20 In addition, there have also been technological advances to care for younger children. In 2015, the first hemodialysis in a neonate with AKI was described using a new equipment, specifically developed for this age group.

The aim of this paper is to review the history and to describe the main information about the epidemiology of AKI in pediatric patients.

## Methods

For this review, we searched the MEDLINE database through the PUBMED portal, using the Mesh term (Medical Subject Headings) "Acute Kidney Injury", selecting the subtopic "Epidemiology". We then applied filters for age (0 to 18 years) and year of publication (last 5 years) were then applied. This search, carried out in April 2017, resulted in 306 papers. We also searched the terms "acute renal failure" and "epidemiology", "acute tubular necrosis" and "epidemiology", in the "title" and "abstract" fields with the same filters (age and year of publication), selecting eight and no paper at all, respectively. The authors evaluated all abstracts, and the papers considered most relevant were examined in their entirety. A second search was carried out in the LILACS database (Latin American and Caribbean Literature in Health Sciences) through the Virtual Health Library portal, using a series of papers on the epidemiology of AKI in Brazil and Latin America, using the following search terms: "acute kidney injury" or "acute renal failure" or "acute renal injury" and the age filter (0 to 18 years). This second search selected 302 papers, which had their summaries examined by the authors. The most relevant papers were evaluated in their entirety; and the selected ones were included in this review. In addition, we carried out direct searches to obtain historical papers on the subject. Papers cited by selected authors and considered relevant were also included in this review.

## AKI Classification

The first standard definition for AKI was the RIFLE classification, published in 2004. It is an acronym with the initials of the five proposed phases for the AKI classification: risk, injury, failure, loss of function, and end-stage renal disease. This classification was based on two widely available markers for renal function: changes in serum creatinine or glomerular filtration rate and urine output.^2^ However, the values considered in its creation contemplated changes in GFR and serum creatinine in adult patients, making it impossible to apply it to the pediatric age group. Within this context, an adaptation of this classification was published in 2007 for the pediatric age group. The P-RIFLE used the estimated reduction in creatinine clearance (CrCl) to measure changes in renal function.^10^

Advances in the studies on the consequences of AKI in critically-ill patients showed that even small increases in serum creatinine caused higher mortality, which led to the development of the Acute Kidney Injury Network (AKIN) for AKI. This new model classified AKI in three stages, according to its severity, with stage 1 defined as an increase of 0.3 mg/dL in serum creatinine in relation to the baseline value.^22^

In 2012, the KDIGO classification was established, aiming to unify the three existing classifications to simplify and universalize its use, since it can be used for adult and pediatric patients. This definition, the most current one available in the literature, also takes into account two easily verified characteristics: serum creatinine (or estimated CrCl for patients under 18 years of age) and urine output.^11^ Although it is the most current and adequate classification for the pediatric age group, there was still a need for adaptation to the neonatal period, a phase in which renal physiology has particularities. Thus, the KDIGO classification for AKI in the neonatal period was published in 2015,^23^ in which stage 2 AKI is when there is reduced urine output for a shorter period; and the absolute value of serum creatinine is greater than or equal to 2.5 mg/dL is considered stage 3, since it represents a CrCl lower than 10 mL/min/1.73 m^2^ in neonates. Another peculiarity is that the baseline creatinine value is defined as the lowest previous value, since creatinine at birth reflects maternal creatinine, and physiologically evolves with falls during the first days of life.^23^

The estimated CrCl calculation is done using the Schwartz formula, which considers that CrCl is the result of the multiplication of the patient's height in centimeters by a constant *k*, divided by serum creatinine in mg/dL.^24^^,^^25^ The original formula uses serum creatinine as measured by the Jaffe method and the k-constant varying according to the patient's age range.^24^ The most current version of the Schwartz formula uses a single value for the constant k = 0.413, regardless of age group and serum creatinine as measured by the enzymatic method.^25^

Estimated creatinine clearance (mL/min/1.73 m^2^) = k X height (cm)/serum creatinine (mg/dL)

Table 1 illustrates the described classifications for AKI.

**Table 1:** AKI available classifications

Classification	Year	Stage	Creatinine	Urinary output
RIFLE^2^	2004	R I F L E	Increase ≥ 1.5x or GFR reduction ≥ 25% Increase ≥ 2x or GFR reduction ≥ 50% Increase ≥ 3 x or creatinine ≥ 4 mg/dL Persistent failure or > 4 weeks Persistent failure for > 3 months	< 0.5mL/kg/h per 6 h < 0.5mL/kg/h per 12 h < 0.3mL/kg/h per 24 h or anuria for 12 h
p-RIFLE^10^	2007	R I F L E	Reduction in the estimated CrCl ≥ 25% Reduction in the estimated CrCl ≥ 50% Reduction in the estimated CrCl ≥ 50% Reduction in the estimated CrCl ≥ 75% or estimated CrCl < 35mL/min/1.73m^2^Persistent failure for > 4 weeks Persistent failure for > 3 months	< 0.5mL/kg/h for 8 h < 0.5mL/kg/h for 16 h < 0.3mL/kg/h for 24 h or anuria for 12 h
AKIN^22^	2007	1 2 3	Increase ≥ 0,3 mg/dL or increase to 150-200% of baseline Increase to 200-300% of baseline Increase to ≥ 300% of baseline or Cr ≥ 4 mg/dL with sharp increase of 0.5 mg/dL	< 0.5mL/kg/h for 6 h < 0.5mL/kg/h for 12 h < 0.3mL/kg/h for 24 h or anuria for 12 h
KDIGO^11^	2012	1 2 3	Increase of 0.3 mg/dL (in 48 h) or 150-200% (in 7 days) Increase ≥ 200-300% Increase ≥ 300%, Cr ≥ 4 mg/dL or dialysis or eGFR < 35mL/min/1.73 m^2^ for < 18 years	< 0.5mL/kg/h for 8 h < 0.5mL/kg/h for 16 h < 0.3mL/kg/h for 24 h or anuria for 12 h
KDIGO neonatal^23^	2015	0 1 2 3	No increase or increase < 0.3 mg/dL Increase ≥ 0.3 mg/dL in 48 h or increase ≥ 1.5-1.9x reference value in 7 days Increase ≥ 2.0-2.9x reference value Increase ≥ 3x reference value or Cr ≥ 2.5 mg/dL or dialysis	≥ 0.5mg/kg/h < 0.5mg/dL for 6-12 h < 0.5mg/dL for ≥ 12 h < 0.3mg/dL for ≥ 24 h or anuria for ≥ 12 h

### Global Epidemiology of Pediatric AKI

Epidemiological data demonstrating the significant financial cost and high morbidity and mortality associated with AKI^26^ have been reported in studies on this subject involving pediatric patients in the literature, in recent years. However, these studies are still concentrated in developed countries. Data on AKI characteristics in developing countries remain scarce.

The first large epidemiological study involving a large number of pediatric patients was published in 2010, using the p-RIFLE for AKI diagnosis. An incidence of AKI of 11% has been demonstrated in patients between 31 days and 21 years of age admitted to a PICU in a single US center. A subsequent multicenter study in the same country described the incidence of 3.9 cases/1,000 hospitalizations and there was a need for ARS in 8.8% of the cases. The authors also reported higher mortality in the group requiring ARS (27.1% versus 14.2% *p* < 0.001).^27^ In a prospective assessment involving 226 children aged 0-14 years submitted to RRT at a single center in New Zealand in the period 2001-2006, the authors reported a mortality rate of 11%.^16^

New studies have been published using the KDIGO classification as a criterion for the diagnosis of AKI. In a retrospective cohort of 8,260 ICU patients, of whom 974 were diagnosed as having AKI according to the KDIGO criteria, 25.3% mortality was observed in 28 days, being higher in patients who did not recover during the observation period (40.5% x 11.2%, p <0.01).^28^ More recently, in a prospective analysis of 4,984 patients between 3 months and 25 years of age, admitted to 32 PICUs in 4 continents, an incidence of 26.9% of AKI at any stage and mortality of 11% in patients with AKI stages 2 or 3 versus 3.4% in patients who did not develop AKI have been reported.^29^ In another large epidemiological study carried out in the United States, published in 2014, the authors assessed preterm extreme low-weight and an incidence of AKI of 39.8% was reported, according to the modified KDIGO classification for the neonatal period, as well as a higher mortality and hospitalization time adjusted for the severity of the patient.^30^

Available studies on the epidemiology of pediatric AKI in developing countries are mostly observational studies carried out in a single center. An exception is a study involving 388,736 patients under the age of 18 years admitted to 27 Chinese hospitals, which reported an incidence of AKI (AKIN) of 0.32% and a mortality rate of 3.4% in patients who developed AKI at any stage.^18^ Studies in Nigeria, India, Thailand and Pakistan showed mortality rates of 41.5%, 50.4% and 30%, respectively.^19^^,^^20^^,^^31^^,^^32^

Data on the worldwide epidemiology of AKI are outlined in Table 2.

**Table 2:** World epidemiology of pediatric AKI

Country	Year	Study design	AKI diagnostic criteria	Patient characteristics	Number of patients	% RRT*	Incidence (%)	Mortality (%)
Nigeria^54^	2004	Prospective Single center	Non-standardized definition	0-15 year-old patients admitted with AKI diagnosis	123	53	---	43.9
Thailand^19^	2006	RetrospectiveSingle center	Non-standardized definition	1 month-17 years patients with AKI diagnosis in Thai hospital	311	17.6	---	41.5 63.6 if RRT
New Zeland^16^	2008	Retrospective Single center	Only patients who required RRT	0-15year-old AKI patients submitted to RRT	226	100	---	11
United States^5^	2010	Retrospective Single center	RIFLE	31 days-21 years patients admitted to the PICU	3.396	1.2	10	30-32 42.5 if RRT
United States^27^	2013	RetrospectiveMulticentric	Patients diagnosed with AKI by the ICD **	0-18 year-old patients admitted to 4121 hospitals of the country in 2009	2.644.263	8.8	0.39	15.3
China^18^	2013	ProspectiveMulticentric	AKIN	15 days to 18 year-old patients admitted to 27 hospitals in 2008	388.736	15.1	0.32	3.4
United States^30^	2014	RetrospectiveSingle center	KDIGO	Newborns with weight ≤ 1500 g admitted to NNICU from 2008-2011	455	0.55	39.8	14.4
United States^28^	2015	Retrospective Single center	KDIGO	1 month-21 year-old patients admitted to the PICU 2003-2012	8260	17.7	11.8	25.3
United States^29^	2017	Prospective Multicentric	KDIGO	3 months-25 year old patients admitted to 32 PICUs in 4 continents	4984	1.5	26.9	5.5 11 if stage 2 or 3
Pakistan^31^	2017	Prospective Single center	pRIFLE	1 month-15 year-old patients with AKI diagnosis admitted in 1 year	116	53	---	5.3
India^32^	2017	Prospective Single center	pRIFLE	2 months-18 year old patients admitted to the PICU in 1 year	380	19	14	36

### Epidemiology of pediatric AKI in Brazil

Studies on the epidemiology of AKI in pediatric patients in Brazil are rare. In a retrospective study published in 2008, a global mortality rate of 53.3% was reported in children aged 0 to 12 years in dialysis because of AKI, who underwent peritoneal dialysis, and it was even higher (73.9%) in the neonatal period.^33^ Another study, published in 2009, selected 110 children from 1 month to 15 years of age, using serum creatinine values above the normal reference for age and height as inclusion criterion, with a lower overall mortality (33.6%), probably due to the fact that patients less than 1 month old were excluded, and also by the inclusion of patients who had not yet reached the most severe stage of AKI.^34^ Studies using pRIFLE for diagnosis and classification of AKI stage in admitted patients in pediatric intensive care units showed that patients who developed AKI during hospitalization had a higher mortality rate and length of hospital stay than patients who maintained normal kidney function.^35^^,^^36^ In a more recent study, limited to sepsis-related AKI, a mortality rate of 33.7% was evidenced. The main risk factors for mortality were duration of hospitalization, use of mechanical ventilation, hypoalbuminemia and the need for dialysis.^37^ In a prospective epidemiological study in which pRIFLE and KDIGO criteria were used for the diagnosis of AKI, a similar prevalence of AKI was found with both (49.4 and 46.2%, respectively).^36^ The observed mortality was 11.4% in patients with AKI diagnosed by pRIFLE and 12.2% in patients diagnosed with AKI according to KDIGO criteria.^38^ Table 3 summarizes the available epidemiological data available about pediatric AKI in Brazil.

**Table 3:** AKI epidemiology in Brazil

Year	Study design	AKI diagnostic criterion	Patient characteristics	Number of patients	% RRT	Incidence	Mortality
2008^33^	RetrospectiveSingle center	Patients submitted to peritoneal dialysis	0-12 year-old patients admitted to the PICU or NNICU who required PD between 2002 and 2006	45	100	---	53.3
2009^34^	Prospective Single center	Creatinine higher than the reference value for age/height	0-15 year-old patients with AKI admitted to the PICU between 2002-2004	110	49.1	8	33.6
2013^35^	Prospective Single center	pRIFLE	28 days-15 year-old patients admitted to the PICU during 3 months	126	12	46	36.2
2015^36^	Retrospective Single center	pRIFLE	29 days-18 year-old patients admitted to the PICU in 1 year	375	---	54,9	16
2016^38^	Prospective Single center	pRIFLE/KDIGO	0-20 years admitted to the PICU in 6 months	160	----	51,3% (pRIFLE) 42% (KDIGO)	11,4% (pRIFLE) 12,2% (KDIGO)
2017^37^	Retrospective Single center	pRIFLE	1 month-11-year old patients admitted to the PICU in 4 years with a diagnosis of sepsis and AKI	77	42.8	---	33.7

### Etiology

The first epidemiological studies on AKI, reported primary kidney disease as a common cause.^39^ With the advent of intensive therapy and technological advances that have improved care for critically ill patients, the etiologies of AKI have changed dramatically. Currently, multifactorial AKI is a reality because, in the intensive care setting, it is common for the same patient to remain exposed, for example, sepsis, shock and drug nephrotoxicity. In addition, complex cardiac surgeries and chemotherapeutic treatment for neoplasia also evolved and became more widely available, leaving these patients also exposed to the risk of AKI related to this health care.

These etiologic changes are most evident in developed countries, where more studies on AKI are available. In one study, involving patients admitted between 1999 and 2001 to a tertiary center in the United States, ischemia was still reported as the leading cause of AKI (21%), followed by nephrotoxicity (16%) and sepsis (11%). These cases occurred prior to the dissemination of the concepts published by the *Surviving Sepsis Campaign*, when the onset of early parenteral antibiotic therapy and volume expansion became recommended as an essential therapy for the reduction of related mortality.^41^ In developed countries, current data point to sepsis and cardiac surgeries as etiologic agents related to AKI in critically ill patients.^27^^,^^42^ When we extend the assessment to hospitalized patients in less complex sectors, the importance of nephrotoxicity as an etiological factor becomes more evident, because although very present in the intensive care setting, its role in the development of AKI is clearly assessed in the absence of other risk factors. Goldstein et al. described the development of AKI in one-third of the low-complexity admissions in patients receiving aminoglycosides for ≥ 3 days or patients who received ≥ 3 nephrotoxic drugs during hospitalization. In neonates, in addition to sepsis, nephrotoxicity and cardiac surgery, perinatal asphyxia also plays an important role as AKI etiology.^44^

In developing countries, primary kidney diseases are still important causes of AKI in the pediatric population. In a multicenter study in China, published in 2013, acute glomerulonephritis was the major cause of AKI in the study population, followed by severe dehydration.^18^

Table 4 highlights the main etiologies of AKI according to the location and year in which the study was carried out.

**Table 4:** AKI etiologies according to the place and year of the study

Year	Study place	Patient characteristics	Main AKI etiologies
2005^40^	United States	0-21 year-old patients diagnosed with AKI.	Ischemia, nephrotoxicity and sepsis
2006^19^	Thailand	0-17 year-old patients diagnosed with AKI	sepsis, hypovolemia and ADG
2007^10^	United States	0-21 year-old patients with AKI.	pneumonia, sepsis and shock
2007^4^	United States	0-25 year-old patients who received Continuous Renal Replacement	sepsis, bone marrow transplant and heart diseases
2008^16^	New Zealand	0-15 year-old patients who received Renal Replacement Therapy	Heart surgery, hemolytic-uremic syndrome and sepsis
2010^42^	Spain	Patients with a mean age of 52 months who received Continuous Renal Replacement Therapy	Heart diseases, sepsis, and renal failure flaring.
2013^27^	United States	0-18 year-old patients admitted to 4,121 hospitals	shock, sepsis and liver diseases
2013^18^	China	0-17 year-old patients admitted to 27 hospitals	Acute glomerulonephritis, severe dehydration and nephrotic syndrome
2016^32^	India	0-18 year-old patients admitted to the PICU in 1 hospital	Shock, sepsis and respiratory failure
2016^31^	Pakistan	0-15 year-old patients admitted in 1 hospital	Post-infectious glomerulonephritis, urolithiasis and crescent GN

### Future perspectives

Currently being developed are extensive research in the search for predictive factors of AKI, which goals are to find factors that can predict or detect risks for the occurrence of AKI, allowing the problem to be avoided or attenuated. The widely used renal function marker, creatinine, is restricted due to its late increase in the course of AKI, as well as its susceptibility to changes by non-renal factors, such as gender, age and muscle mass.^45^ The new biomarkers appeared as a large promise in this regard. Among them, the most widely studied is neutrophil gelatinase-associated lipocalin (NGAL), which showed good accuracy for the detection of AKI after post-operative insult in cardiac surgeries, sepsis and contrast use.^46^^-^49

**Figure 1:**
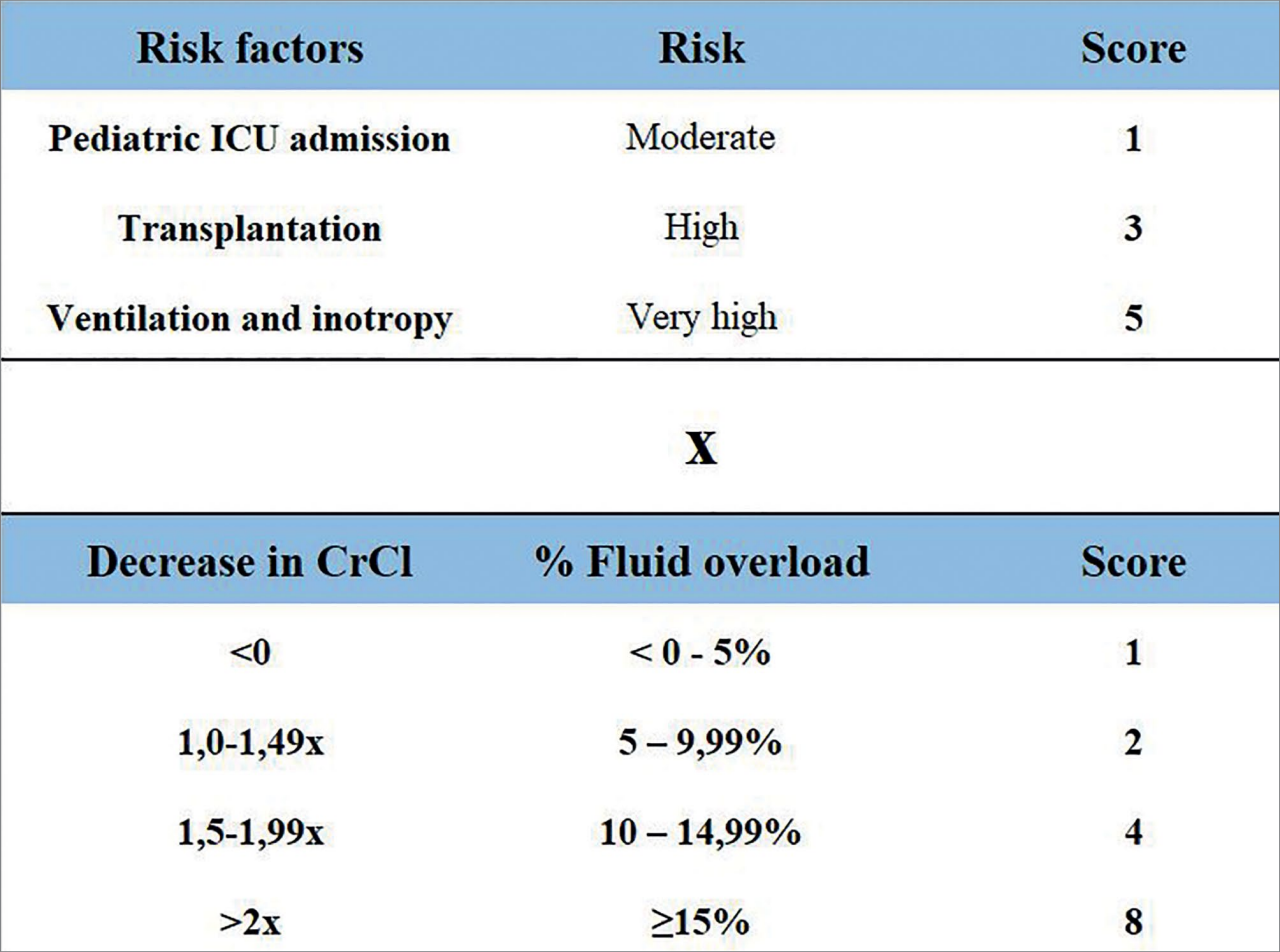
Renal Angina Index calculation. Note: The result may vary between 1 and 40. Values ≥ 8 establishes the presence of renal angina.

Source: translated and adapted from Basu *et al*.^50^

In order to improve the pre-test probability of available biomarkers in 2010, the concept of "renal angina" was developed, which uses features that indicate risk for AKI and early clinical signs of renal damage for its calculation, creating a predictor score of AKI, and which could serve as a screening tool to determine which patients should have their biomarkers dosed.^50^^,^^51^ For the calculation of the "Renal Angina Index" (RAI), the authors defined factors that make the child susceptible to AKI, and early clinical signs of AKI (injury). The presence of each characteristic assigns a score, and the score obtained in "risk" is multiplied by the score obtained in "injury", resulting in the "Renal Angina Index". A result greater than or equal to 8 showed prediction for AKI on the 3rd day of admission with an area under the curve of 0.74-0.81.^52^ Figure 1 illustrates how the RAI is calculated.

In a recent prospective study, urinary NGAL was incorporated into the RAI and they found that the combined model was able to predict severe and persistent AKI (KDIGO 2 or 3), with an area under the curve of 0.97.^53^ Although promising, these observations need to be replicated in other locations.

## Conclusion

The AKI is a serious condition, with a multifactorial etiology in many cases, and with variable mortality, reaching more than 60% in patients undergoing dialysis. From the epidemiological point of view, there is still a significant lack of robust studies on the incidence, prevalence and outcomes of AKI in the pediatric population, notably in developing countries, such as ours. Promising studies aimed at early diagnosis and intervention may prevent its occurrence or mitigate its effects.
